# Enhanced Expression of WD Repeat-Containing Protein 35 via Nuclear Factor-Kappa B Activation in Bupivacaine-Treated Neuro2a Cells

**DOI:** 10.1371/journal.pone.0086336

**Published:** 2014-01-21

**Authors:** Lei Huang, Fumio Kondo, Misako Harato, Guo-Gang Feng, Naoshisa Ishikawa, Yoshihiro Fujiwara, Shoshiro Okada

**Affiliations:** 1 Department of Pharmacology, Aichi Medical University School of Medicine, Nagakute, Aichi, Japan; 2 Department of Anesthesiology, Aichi Medical University School of Medicine, Nagakute, Aichi, Japan; University of Missouri-Kansas City, United States of America

## Abstract

The family of WD repeat proteins comprises a large number of proteins and is involved in a wide variety of cellular processes such as signal transduction, cell growth, proliferation, and apoptosis. Bupivacaine is a sodium channel blocker administered for local infiltration, nerve block, epidural, and intrathecal anesthesia. Recently, we reported that bupivacaine induces reactive oxygen species (ROS) generation and p38 mitogen-activated protein kinase (MAPK) activation, resulting in an increase in the expression of WD repeat-containing protein 35 (WDR35) in mouse neuroblastoma Neuro2a cells. It has been shown that ROS activate MAPK through phosphorylation, followed by activation of nuclear factor-kappa B (NF-κB) and activator protein 1 (AP-1). The present study was undertaken to test whether NF-κB and c-Jun/AP-1 are involved in bupivacaine-induced WDR35 expression in Neuro2a cells. Bupivacaine activated both NF-κB and c-Jun in Neuro2a cells. APDC, an NF-κB inhibitor, attenuated the increase in NF-κB activity and WDR35 protein expression in bupivacaine-treated Neuro2a cells. GW9662, a selective peroxisome proliferator-activated receptor-γ antagonist, enhanced the increase in NF-κB activity and WDR35 protein expression in bupivacaine-treated Neuro2a cells. In contrast, c-Jun siRNA did not inhibit the bupivacaine-induced increase in WDR35 mRNA expression. These results indicate that bupivacaine induces the activation of transcription factors NF-κB and c-Jun/AP-1 in Neuro2a cells, while activation of NF-κB is involved in bupivacaine-induced increases in WDR35 expression.

## Introduction

The family of WD repeat (WDR) proteins comprises a large number of proteins and is involved in a wide variety of cellular processes such as signal transduction, cell growth, proliferation, and apoptosis [1], [2]. WD repeat-containing protein 35 (WDR35) is a novel member of the WDR protein family [3]. We reported that enhanced WDR35 expression may mediate apoptosis in the kidneys of streptozotocin-induced diabetic rats [4], in the livers of lipopolysaccharide (LPS)-treated rats [5], and in the hippocampus of domoic acid-treated rats [6]. In a mouse mutation screen for developmental phenotypes, Mill et al. [7] identified a mutation in the WDR35 gene as a cause of defects in cilia formation and function, resulting in midgestation lethality associated with abnormalities characteristic of defects in the Hedgehog signaling pathway.

Reactive oxygen species (ROS) are known to stimulate a number of events and pathways that lead to cell death, including mitogen-activated protein kinase (MAPK) signal transduction pathways [8]. In neuronal cells, p38 MAPK, a member of the MAPK family, is preferentially activated by environmental stress and inflammatory cytokines, and it has been shown to promote neuronal cell death [9]. Bupivacaine is a sodium channel blocker administered for local infiltration, nerve block, epidural, and intrathecal anesthesia [10]. Bupivacaine-induced neurotoxicity has been associated with the generation of ROS [11] and activation of p38 MAPK [12], [13]. Recently, we demonstrated that bupivacaine induces ROS generation and p38 MAPK activation, resulting in apoptosis in mouse neuroblastoma Neuro2a cells [14]. Bupivacaine also increased WDR35 expression in a dose- and time-dependent manner; however, blocking upregulation of WDR35 expression with WDR35 siRNA in Neuro2a cells had no effect on the increase in cell death induced by bupivacaine [14]. These results prompted us to further investigate signaling downstream of p38 MAPK pathways responsible for up-regulating WDR35 expression in bupivacaine-treated Neuro2a cells.

Transcription factors such as nuclear factor-kappa B (NF-κB) and activator protein 1 (AP-1) are implicated in the inducible expression of a wide variety of genes involved in oxidative stress and cellular response mechanisms [8], [15]–[17]. Many observations indicate that p38 MAPK can stimulate NF-κB and AP-1 signaling through diverse mechanisms [18]–[21]. Cocaine has been shown to activate NF-κB and increase the expression of brain derived neurotrophic factor in PC-12 cells [22]. Bupivacaine remarkably upregulated the expression of c-Jun, the most potent transcription factor of the AP-1 family, in HL-60 cells [23]. Although these reports indicate that local anesthetics activate NF-κB and c-Jun/AP-1, the relevance of these transcription factors to WD repeat protein expression has not been fully investigated. Very recently, Teng et al. [24] reported that NF-κB binds to the Leucine-rich repeats and WD repeat domain containing 1 (LRWD1) promoter and regulates its activity in a human testicular embryonal carcinoma cell line. The present study demonstrates that bupivacaine activates both NF-κB and c-Jun/AP-1 in Neuro2a cells, while only NF-κB is involved in bupivacaine-induced increases in WDR35 expression.

## Materials and Methods

### Cell Culture

Mouse neuroblastoma Neuro2a cells were purchased from the Health Science Research Resources Bank (Tokyo, Japan). The cells were maintained in RPMI-1640 medium (Sigma-Aldrich, St. Louis, MO, USA) containing 10% fetal bovine serum with 100 units/ml penicillin and 100 g/ml streptomycin (Gibco BRL, Grand Island, NY, USA). The cells were maintained at 37°C in a humidified atmosphere with 5% CO_2_. The culture medium was replaced every 2–3 days. To prepare cell suspensions, the cells were treated with trypsin (0.25%)-EDTA (1 mM) (Gibco BRL, Grand Island, NY, USA), transferred to a 6-cm culture plate at a density of 1.5×10^6^ cells per dish, and cultured overnight.

In our previous study, we reported that bupivacaine dose-dependently increased WDR35 expression and that maximal WDR35 expression was observed with a concentration of 2 mM bupivacaine at 9 h [14]. As the maximal effect was reached at 9 h, a time point of 9 h of bupivacaine treatment was used for the following experiments. Since higher concentrations than 2 mM induced cell death, we used 2 mM of bupivacaine in the present study. In addition, 2 mM bupivacaine decreased cell viability of Neuro2a cells, resulting in a 70% decrease in cell viability after an incubation of 9 h (data not shown).

### Western Blot Analysis

Nuclear or cytoplasmic extracts were prepared by the use of NE-PER Nuclear and Cytoplasmic Extraction Reagents (Pierce, Rockford, IL, USA) according to the manufacturer’s instructions. Protein concentration was determined by a DC protein assay kit (Bio-Rad, Richmond, CA, USA). Proteins were separated by SDS-PAGE and transferred to PVDF membranes (Immobilon-P, Millipore, Bedford, MA, USA). These membranes were probed with anti-WDR35 peptide antibody (amino acids 459–473, 1∶500), which was designed, produced, and purified by Medical & Biological Laboratories (Nagoya, Japan), or with antibodies against c-Jun, phospho-c-Jun (Ser73) (P-c-Jun), phospho-IκB-α (P-IκB-α), NF-κB (p65), α-tubulin, or nucleolin (Cell Signaling Technology, Danvers, MA, USA; 1∶1000). Detection was performed with the Western blotting reagent ECL Prime (GE Healthcare, Buckinghamshire, UK). Protein levels were quantified by densitometric scanning with the Gel-Pro Analyzer (Media Cybernetics, Inc., USA) and expressed as the ratio to α-tubulin or nucleolin levels as described previously [4], [14].

### NF-κB DNA Binding

Neuro2a cells were treated with bupivacaine (2 mM) for 9 h. The nuclear fraction was then collected from cells using the Active Motif nuclear kit (Active Motif North America, Carlsbad, CA, USA) according to the manufacturer’s instructions. NF-κB DNA binding activity in the nuclear fraction was measured using the Active Motif NF-κB family kit according to the manufacturer’s instructions.

### Quantitative Real-time Polymerase Chain Reaction (qPCR) Analysis

Total RNA (1 µg) was extracted from cultured Neuro2a cells with TRIzol® reagent (Invitrogen, Carlsbad, CA, USA) and reverse transcribed with the ReverTra Ace® qPCR RT kit (Toyobo, Osaka, Japan). qPCR was performed with the ABI StepOne Plus real-time PCR system and a TaqMan Gene Expression Assay (Applied Biosystems, Tokyo, Japan) according to the manufacturer’s instructions. The primers and TaqMan MGB probe for mouse c-Jun (Mm00495062_s1) and WDR35 (Mm00552650_m1) were purchased from Applied Biosystems. The amount of c-Jun or WDR35 PCR product was calculated relative to the internal control beta-actin (ACTB, Mm00607939_s1; Applied Biosystems), glyceraldehyde-3-phosphate dehydrogenase (GAPDH, Mm99999915_g1), or 18S ribosomal RNA (18S rRNA, Mm03928990_g1), and was compared between experimental and control groups by the ΔΔC_T_ method, as reported previously [3], [14]. The difference of internal control did not affect the results in qPCR experiments.

### Small Interfering RNA (siRNA) Transfection

Neuro2a cells were transfected with 5 nM c-Jun siRNA (siRNA ID: s201552; Ambion, Austin, TX, USA), 5 nM WDR35 siRNA (siRNA ID: s93029; Ambion), or 10 nM control siRNA (negative control #1 siRNA, catalog no. 4390843; Ambion) using Lipofectamine RNAiMAX (Invitrogen) according to the manufacturer’s instructions. Effects of siRNA on the expression of c-Jun or WDR35 mRNA were tested with qPCR after 24 h of transfection.

### Statistical Analysis

All results are expressed as the mean ± standard error of the mean (SEM). Data were analyzed with one-way analysis of variance (ANOVA), and significant differences between treatments were assessed by use of Tukey’s test. Differences were considered significant at *P*<0.05.

## Results

### Bupivacaine Induces NF-κB Activation in Neuro2a Cells

**Figure 1:**
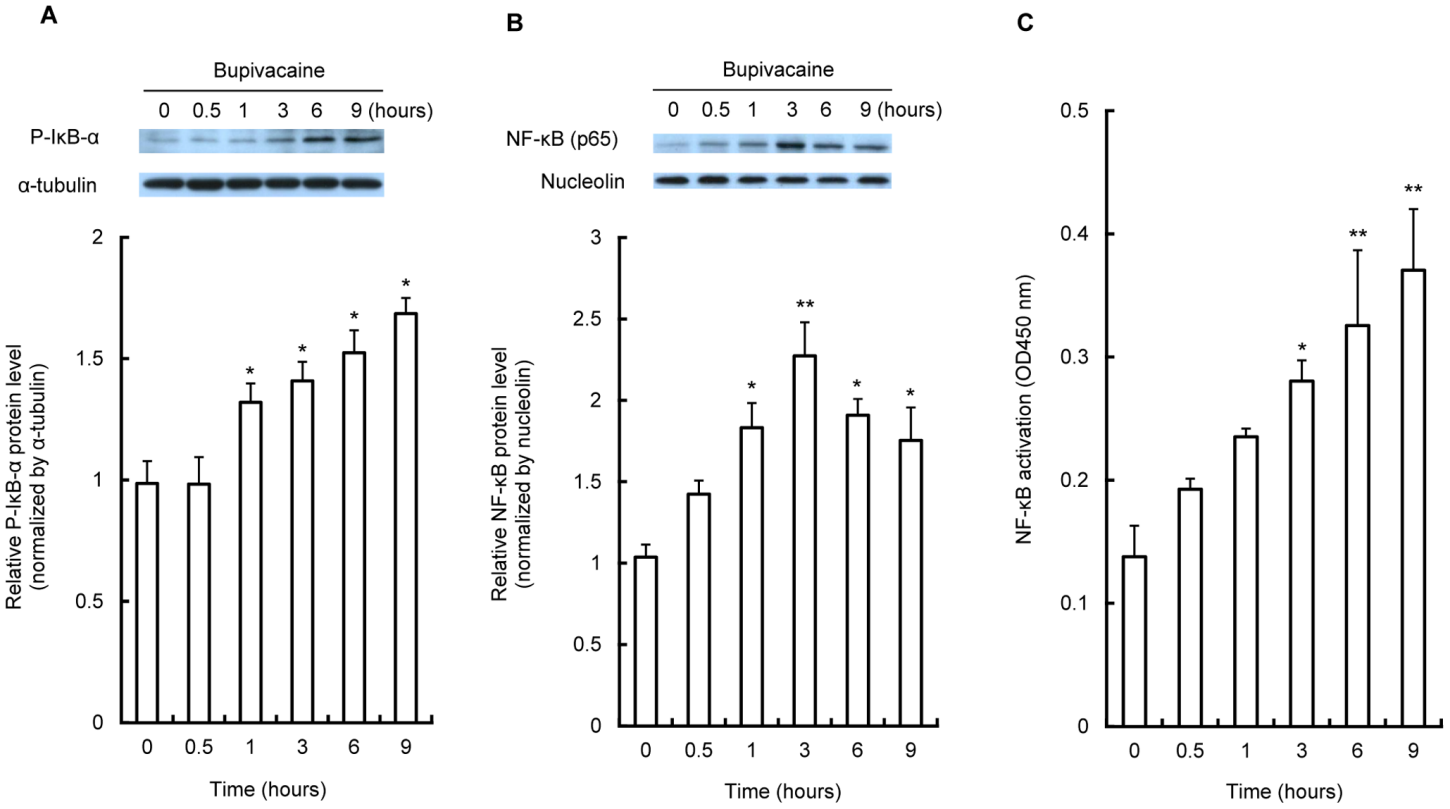
Effect of bupivacaine on cytosolic phosphorylation of IκB-α, nuclear NF-κB expression, and NF-κB activation. Neuro2a cells were treated with 2(A) Cytoplasmic protein expression of phospho-IκB-α (P-IκB-α) and (B) nuclear protein expression of NF-κB (p65) were measured by Western blotting. Cytosolic protein α-tubulin and nuclear protein nucleolin were detected as protein loading controls. (C) The nuclear fraction was collected and NF-κB DNA binding activity in the nuclear fraction was measured using the Active Motif NF-κB family kit. *P<0.05 and **P<0.01 vs. control (not treated with bupivacaine, n = 3).

Activation of NF-κB is triggered by phosphorylation and subsequent degradation of inhibitor of κB (IκB). This process subsequently leads to translocation of the free NF-κB to the nucleus to activate transcription of specific target genes [25]. To determine whether NF-κB is activated in bupivacaine-treated Neuro2a cells, we first examined the protein expression of cytosolic phospho-IκB-α (P-IκB-α) and nuclear NF-κB (p65) in 2 mM bupivacaine-treated Neuro2a cells by Western blotting. As shown in Figure 1A, treatment of Neuro2a cells with bupivacaine significantly increased phosphorylation levels of IκB-α in the cytoplasm at 1 to 9 h (*P*<0.05 at 1 h and later). In addition, bupivacaine significantly increased nuclear NF-κB (p65) protein expression at 1 to 9 h (*P*<0.05 at 0.5, 6 and 9 h and *P*<0.01 at 3 h; Figure 1B).

Next, NF-κB DNA binding activity in the nuclear fraction was measured. Treatment of Neuro2a cells with 2 mM bupivacaine significantly increased NF-κB activity at 3 to 9 h (*P*<0.05 at 3 h and *P*<0.01 at 6 h and later; Figure 1C).

### APDC, an NF-κB Inhibitor, Attenuates the Increase in NF-κB Activity and WDR35 Protein Expression in Bupivacaine-treated Neuro2a cells

**Figure 2:**
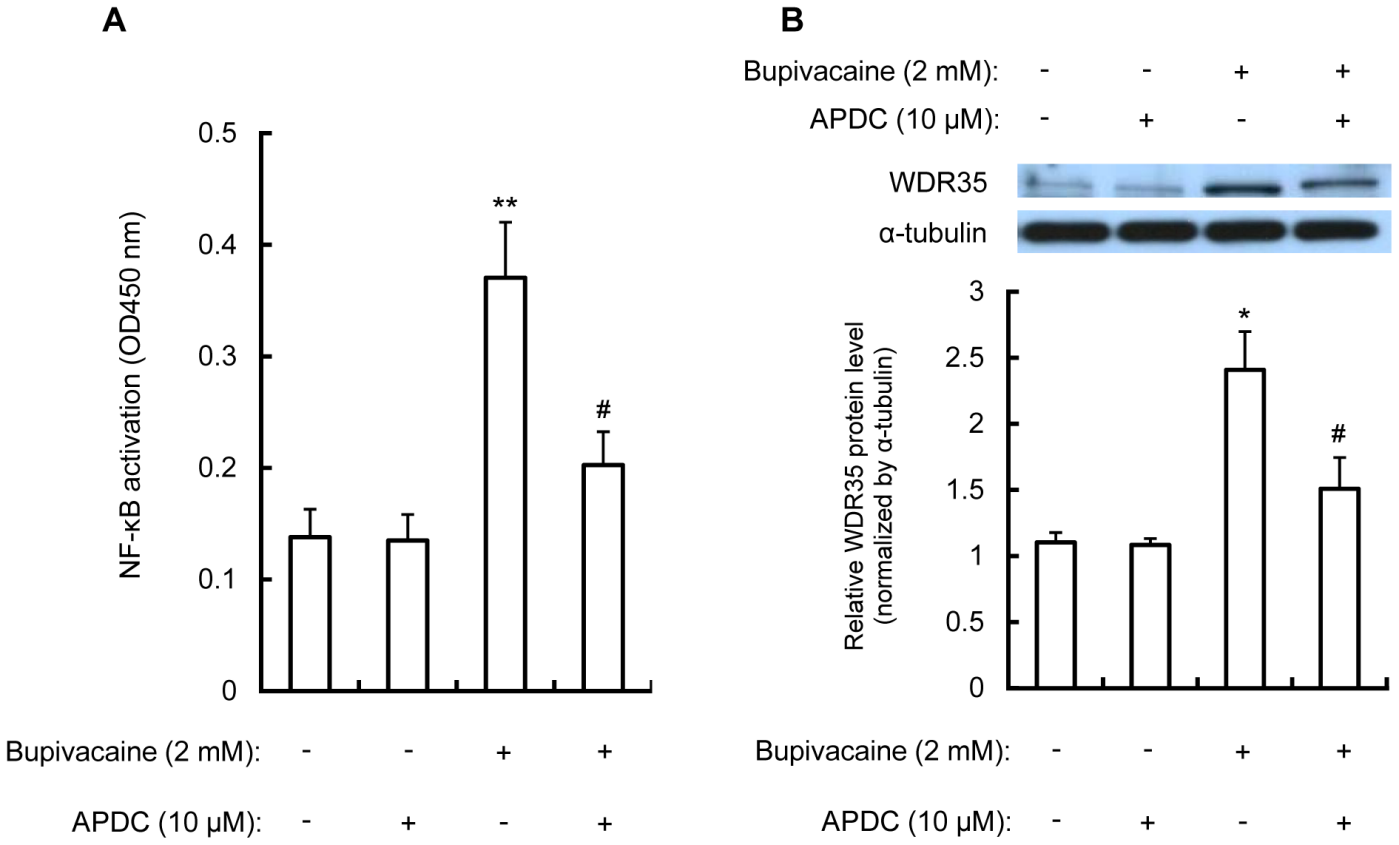
Effect of APDC on bupivacaine-induced NF-κB activity and WDR35 protein expression. Neuro2a cells were treated with NF-κB inhibitor APDC (10 µM) for 1 h, followed by bupivacaine (2 mM) for 9 h. (A) The nuclear fraction was collected and NF-κB DNA binding activity in the nuclear fraction was measured using the Active Motif NF-κB family kit. (B) WDR35 protein expression in the cytoplasm was measured by Western blotting. Cytosolic protein α-tubulin was detected as a protein loading control. *P<0.05 and **P<0.01 vs. control (not treated with bupivacaine), ^#^ *P*<0.05 vs. absence of APDC (n = 3).

APDC is a widely used NF-κB inhibitor in addition to an antioxidant [26]–[28]. Neuro2a cells were treated with APDC (10 µM; Calbiochem, La Jolla, CA, USA) for 1 h, followed by bupivacaine (2 mM) for 9 h. As shown in Figure 2A, APDC significantly attenuated the bupivacaine-induced increase in NF-κB activity in the nuclear fraction (*P*<0.05).

The effect of APDC on bupivacaine-induced WDR35 protein expression in the cytoplasm in Neuro2a cells was measured. As shown in Figure 2B, treatment with APDC significantly attenuated the bupivacaine-induced increase in WDR35 protein expression (*P*<0.05). In contrast, WDR35 protein expression in the nuclear fraction was not observed throughout the experiment (data not shown).

### GW9662, a Selective Peroxisome Proliferator-activated Receptor-γ (PPARγ) Antagonist, Enhances the Increase in NF-κB Activity and WDR35 Protein Expression in Bupivacaine-treated Neuro2a cells

**Figure 3:**
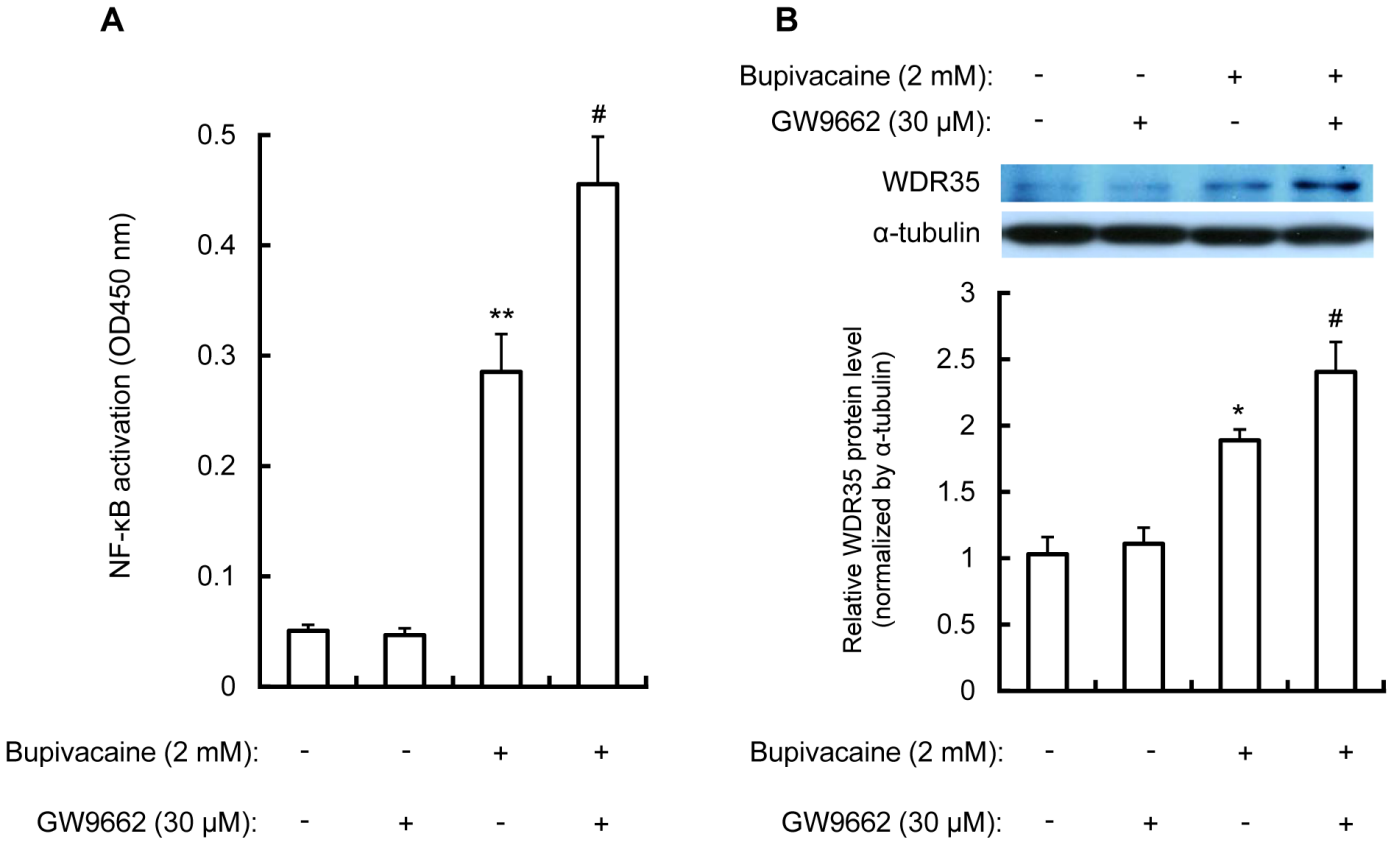
Effect of GW9662 on bupivacaine-induced NF-κB activity and WDR35 protein expression. Neuro2a cells were treated with the PPARγ antagonist GW9662 (30 µM) for 16 h, followed by bupivacaine (2 mM) for 9 h. (A) The nuclear fraction was collected and NF-κB DNA binding activity in the nuclear fraction was measured using the Active Motif NF-κB family kit. (B) WDR35 protein expression in the cytoplasm was measured by Western blotting. Cytosolic protein α-tubulin was detected as a protein loading control. *P<0.05 and **P<0.01 vs. control (not treated with bupivacaine), ^#^ *P*<0.05 vs. absence of GW9662 (n = 3).

Nuclear receptor PPARγ is a ligand-activated transcription factor that plays an important role in regulating NF-κB-induced responses via inhibiting the activation of NF-κB [29], [30]. Since GW9662, a selective PPARγ antagonist, was shown to activate NF-κB in neuronal cells [31], [32], we further investigated the effects of GW9662 on WDR35 expression in bupivacaine-treated Neuro2a cells. Neuro2a cells were treated with GW9662 (30 µM; Cayman Chemicals, Ann Arbor, MI, USA) for 16 h, followed by bupivacaine (2 mM) for 9 h. As shown in Figure 3A, GW9662 significantly enhanced the bupivacaine-induced increase in NF-κB activation in the nuclear fraction (*P*<0.05).

The effect of GW9662 on bupivacaine-induced WDR35 protein expression in the cytoplasm in Neuro2a cells was also measured. As shown in Figure 3B, treatment with GW9662 significantly enhanced the bupivacaine-induced increase in WDR35 protein expression (*P*<0.05).

### Bupivacaine Increases the Expression of c-Jun in Neuro2a Cells

AP-1 is a transcription factor associated with cell proliferation, survival, differentiation, apoptosis, and stress responses [17]. AP-1 is a dimer most commonly formed by the combination of structurally and functionally related members of the Jun protein family (c-Jun, JunB, and JunD) and the Fos protein family (c-Fos, FosB, Fra-1, and Fra-2). Activation of the MAPK pathway has been shown to regulate transcription factor c-Jun/AP-1 activation by oxidative stimuli [16], [20], [21], [33], [34]. Phosphorylation of c-Jun at serine 63 and/or 73 accelerates c-Jun translocation into the nucleus and enhances AP-1 transcriptional activity 17,35.

**Figure 4:**
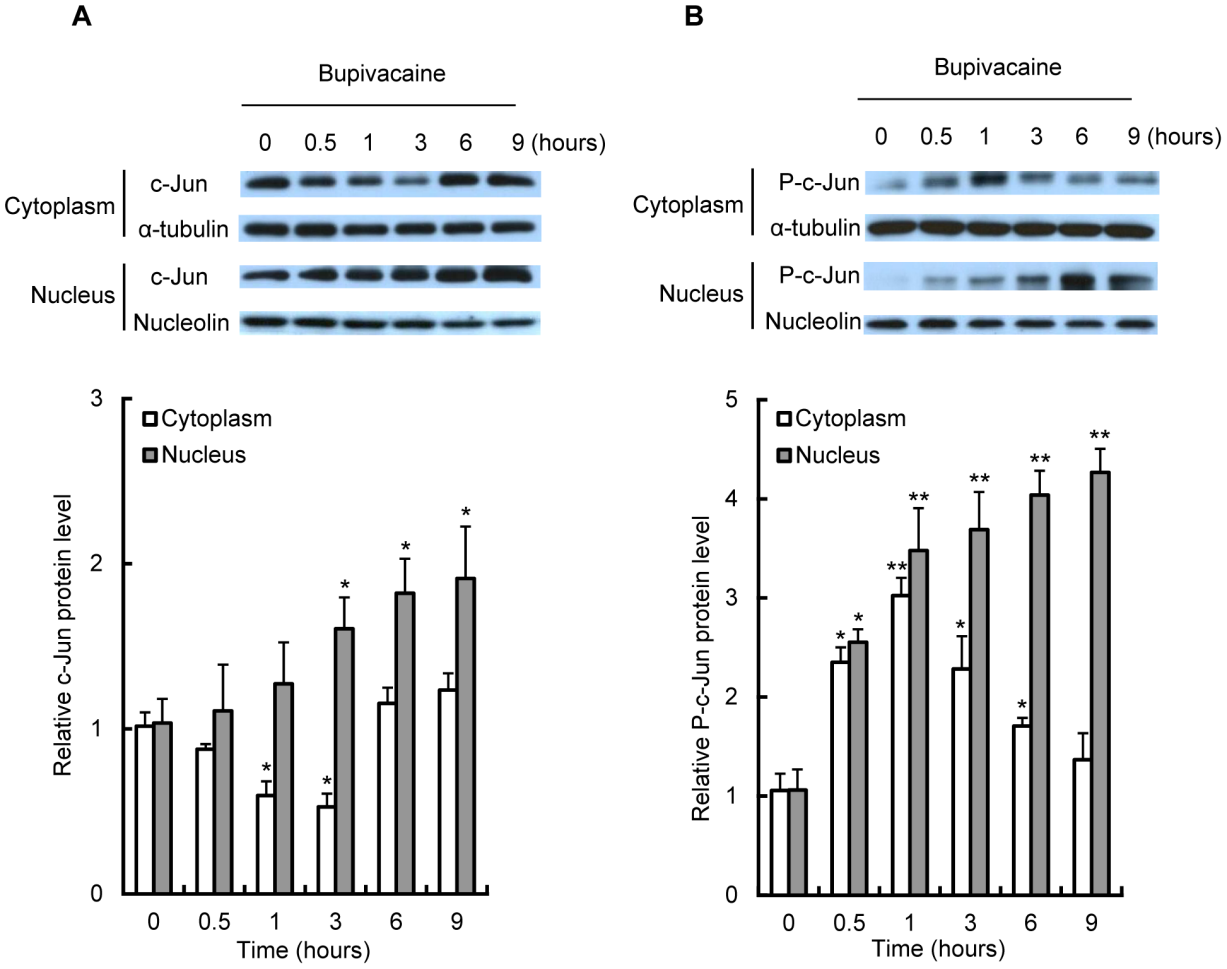
Effect of bupivacaine on expression of c-Jun and phosphorylation of c-Jun in cytoplasm and nucleus. Neuro2a cells were treated with 2(A) and nuclear (B) protein expression of c-Jun and phospho-c-Jun (P-c-Jun) following cell exposure to bupivacaine (2 mM) was measured by Western blotting. Cytosolic protein α-tubulin and nuclear protein nucleolin were detected as protein loading controls. *P<0.05 and **P<0.01 vs. control (not treated with bupivacaine, n = 3).

To determine whether c-Jun is activated in bupivacaine-treated Neuro2a cells, we examined c-Jun protein expression in cytoplasmic and nuclear fractions in 2 mM bupivacaine-treated Neuro2a cells. As shown in Figure 4A, c-Jun protein expression in the cytoplasm was significantly decreased by bupivacaine at time points from 1 to 3 h (*P*<0.05) and then returned to control levels at 6 h. In contract, bupivacaine significantly increased c-Jun protein expression in the nuclear fraction at time points from 3 to 9 h (*P*<0.05).

As shown in Figure 4B, treatment with 2 mM bupivacaine significantly increased phosphorylation levels of c-Jun (P-c-Jun) in the cytoplasm at 0.5 h, which was maintained up to 1 h and then slowly decreased to control levels (*P*<0.05 at 0.5, 3, and 6 h and *P*<0.01 at 1 h). Furthermore, bupivacaine significantly increased phosphorylation levels of c-Jun in the nuclear fraction at time points from 0.5 to 9 h (*P*<0.05 at 0.5 h and *P*<0.01 at 1 h and later).

### c-Jun siRNA does not Inhibit the Bupivacaine-induced Increase in WDR35 mRNA Expression in Neuro2a cells

**Figure 5:**
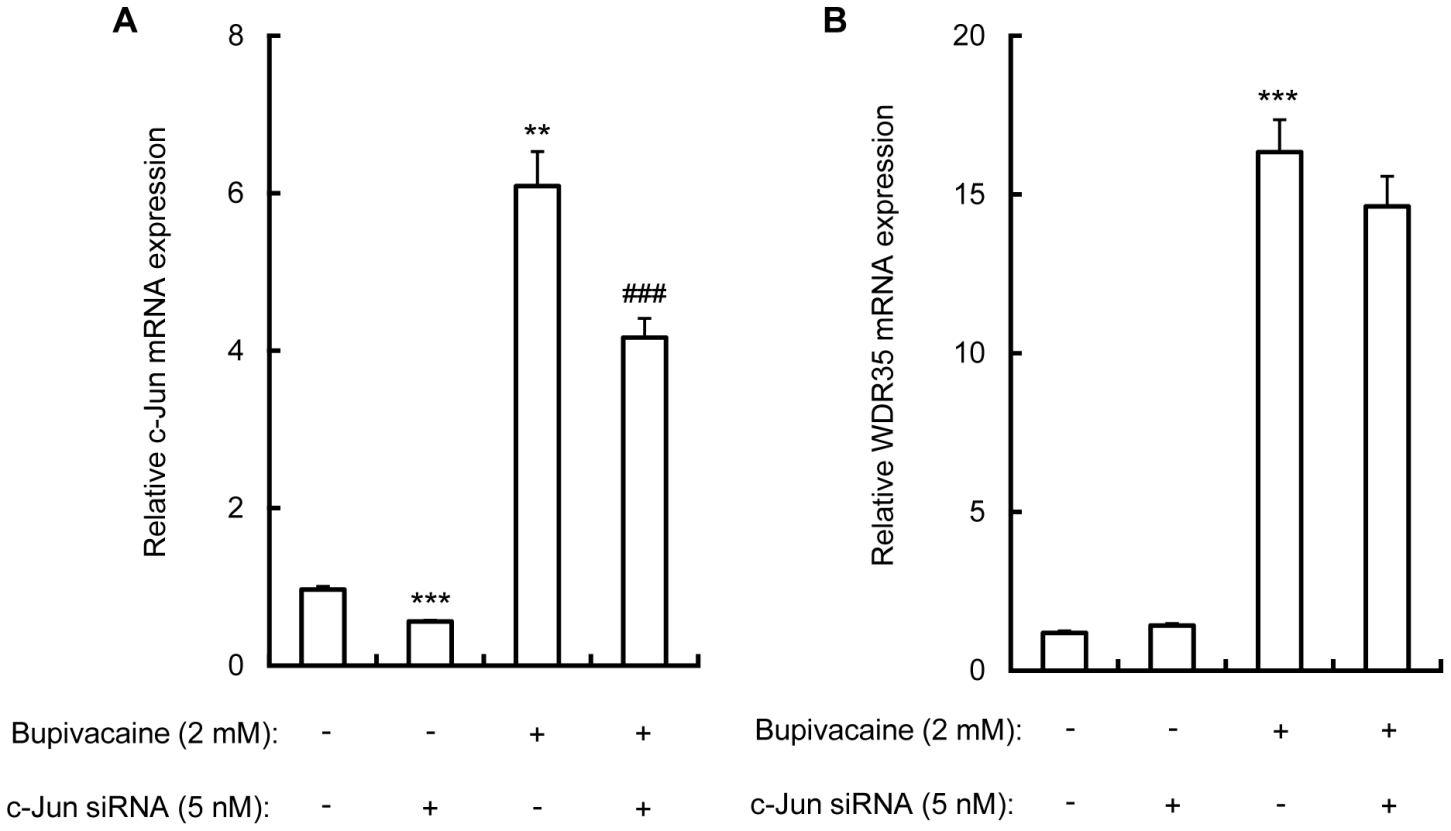
Effect of c-Jun siRNA on bupivacaine-induced WDR35 mRNA expression. Neuro2a cells were transfected with c-Jun siRNA (5 nM) for 24 h, then bupivacaine (2 mM) was added and the cells were incubated for another 9 h. c-Jun (A) and WDR35 (B) mRNA expression was analyzed by qPCR and expressed relative to the expression of ACTB mRNA, respectively. **P<0.01 and ***P<0.001 vs. control (not treated with siRNA or bupivacaine), ^###^ *P*<0.001 vs. treated with bupivacaine alone (n = 4).

The effect of c-Jun siRNA on bupivacaine-induced WDR35 mRNA expression in Neuro2a cells was measured. Cells were transfected with c-Jun siRNA (5 nM) for 24 h, then bupivacaine (2 mM) was added. Cells were incubated for another 9 h, and the expression of c-Jun and WDR35 mRNA was analyzed by qPCR and expressed relative to the expression of ACTB mRNA. Although transfection of Neuro2a cells with c-Jun siRNA significantly attenuated the bupivacaine-induced increase in c-Jun mRNA expression (*P*<0.001; Figure 5A), it did not inhibit the increase in WDR35 mRNA expression (Figure 5B). WDR35 siRNA or control siRNA had no effect on the expression of c-Jun mRNA in bupivacaine-treated Neuro2a cells (data not shown).

## Discussion

Recently, we demonstrated that bupivacaine induces ROS generation and p38 MAPK activation, resulting in an increase in expression of WDR35 in Neuro2a cells [14]. In this study, we provided the first evidence that activation of NF-κB is involved in bupivacaine-induced increases in WDR35 expression in Neuro2a cells. Bupivacaine induced activation of the transcription factors NF-κB and c-Jun in Neuro2a cells. The bupivacaine-induced increase in WDR35 protein expression was attenuated by the NF-κB inhibitor APDC and was enhanced by the PPARγ antagonist GW9662. The increase in WDR35 mRNA expression was not inhibited by c-Jun siRNA. These results suggest that NF-κB is an important transcription factor acting downstream of p38 MAPK pathways responsible for up-regulating WDR35 expression in bupivacaine-treated Neuro2a cells.

We found that bupivacaine increased phosphorylation levels of IκB-α in the cytoplasm and NF-κB protein expression in the nucleus, leading to increases in NF-κB activation in the nucleus. To determine whether NF-κB activation is involved in bupivacaine-induced WDR35 expression, we examined the effects of APDC, a widely used NF-κB inhibitor in addition to an antioxidant [26]–[28], on WDR35 expression in bupivacaine-treated Neuro2a cells. APDC treatment resulted in an attenuation of the bupivacaine-induced increase in WDR35 expression in Neuro2a cells. When PPARγ was inhibited with GW9662, the suppressed nuclear translocation of NF-κB p65 and NF-κB DNA binding activity were attenuated [31], [32]. GW9662 treatment increased the bupivacaine-induced increase in NF-κB activation, resulting in an increase in WDR35 expression. Furthermore, other research groups have reported that the promoter region of LRWD1, another WDR protein family member, was also regulated by NF-κB [24]. Collectively, these results indicate that bupivacaine-induced increases in WDR35 expression are transcriptionally regulated by NF-κB.

In the present study, we showed that bupivacaine induces phosphorylation of c-Jun in the cytoplasm, leading to increases in phosphorylated c-Jun in the nucleus. Previous reports also showed that bupivacaine upregulated the expression levels of NF-κB and c-Jun genes in HL-60 cells [23]. These results indicate that bupivacaine activates not only NF-κB but also c-Jun in Neuro2a cells. We next examined the involvement of c-Jun in WDR35 expression in bupivacaine-treated Neuro2a cells. Interestingly, blocking upregulation of c-Jun mRNA expression with c-Jun siRNA in Neuro2a cells had no effect on the bupivacaine-induced increase in WDR35 mRNA expression. These results suggest that WDR35 expression is strictly regulated by NF-κB independently of c-Jun.

Recent studies demonstrated that the WDR35 gene is involved in several human diseases such as type 2 diabetes [36], acute lymphoblastic leukemia [37], coronary artery disease [38], and Sensenbrenner syndrome [39]. We previously reported that WDR35 activates caspase-3 and promotes tumor necrosis factor (TNF)-α−induced apoptosis in HEK293 cells [3]. We also reported that enhanced expression of WDR35 may mediate the activation of caspase-3 through a mitochondrial signaling pathway in LPS-induced hepatocyte apoptosis [5]. However, the transcriptional regulation of WDR35 expression has not been fully elucidated. In the present study, we provided the first evidence of the sequence of events for bupivacaine-induced WDR35 expression by biochemical and pharmacological examinations *in vitro*, in which NF-κB is an essential transcription factor for up-regulating WDR35 expression. Taken together with our previous study, bupivacaine was found to induce ROS production, p38 MAPK phosphorylation, and NF-κB activation, resulting in an increase in the expression of WDR35 in Neuro2a cells. Although the function of WDR35 is still unknown, other WDR proteins have been shown to be components for the assembly of signaling complexes [40] and to act as scaffolding proteins [41]. Further studies incorporating proteome analyses of proteins interacting with WDR35 will help to understand the role of WDR35.

In conclusion, our results indicate that bupivacaine activates both NF-κB and c-Jun/AP-1 in Neuro2a cells, while only NF-κB is involved in bupivacaine-induced increases in WDR35 expression.
